# Selective Antibiofilm Effects of *Lucilia sericata* Larvae Secretions/Excretions against Wound Pathogens

**DOI:** 10.1155/2014/857360

**Published:** 2014-06-11

**Authors:** Jana Bohova, Juraj Majtan, Viktor Majtan, Peter Takac

**Affiliations:** ^1^Institute of Zoology, Slovak Academy of Sciences, 845 06 Bratislava, Slovakia; ^2^Department of Microbiology, Faculty of Medicine, Slovak Medical University, 833 03 Bratislava, Slovakia; ^3^Scientica s.r.o., 831 06 Bratislava, Slovakia

## Abstract

*Background*. Maggot debridement therapy (MDT), using *Lucilia sericata* larvae, represents efficient, simple, and low-cost therapy for the treatment of chronic wounds. *Aim*. The aim was to investigate the antibiofilm activity of maggot excretions/secretions (ES) against biofilm of wound isolates *Staphylococcus aureus* (*S. aureus*), *Enterobacter cloacae* (*E. cloacae*), and *Proteus mirabilis* (*P. mirabilis*). *Methods*. Quantification of biofilm formation, was carried out using a microtiter plate assay. Proteolytic activity of maggot ES was performed using skim milk agar plates. A solid phase extraction and reverse phase HPLC C18 chromatography were employed to the isolate of maggot ES antibiofilm compounds. *Results*. Maggot ES at 100 mg/mL concentration significantly reduced biofilm formation thus disrupting established biofilm of *E. cloacae*. Heat-treated ES did not show any antibiofilm activity towards *E. cloacae*. Similar results were obtained in the case of *S. aureus*; however, the heat-treatment of maggot ES did not affect its antibiofilm activity. Moreover, a compound with molecular weight of 25 kDa exhibiting antibiofilm activity was identified in maggot ES. On the other hand, maggot ES protected and even stimulated *P. mirabilis* biofilm formation. *Conclusions*. Our results suggest that maggot ES may act selectively against different bacterial strain.

## 1. Introduction

It has been known for decades that some chronic bacterial infections are caused by the ability of bacteria to form biofilm [1–7]. The classic example of biofilm involvement in chronic infections is nonhealing dermal wounds. Biofilm growth and its persistence within wounds have recently been suggested as being a contributing factor towards impaired healing [3, 8]. Using cultural and molecular techniques, it has been shown that pathogenic biofilm in chronic wound is generally polymicrobial, with certain species such as* Pseudomonas aeruginosa *(*P. aeruginosa*) and* Staphylococcus aureus* (*S. aureus*) which are often predominant. Recently, several studies have demonstrated that a wide variety of bacteria with different physiological and phenotypic preferences are common as part of the pathogenic biofilm communities in chronic wounds [9–12]. There is an estimated average of 6.3 bacterial species in chronic ulcers [13]. Chronic wounds associated with biofilm are a worldwide problem joined with high economical costs, social and psychological deprivation, and pain. The treatment of bacterial biofilm in wound is complicated due to the underlying mechanisms of biofilm growth. Furthermore, mixed species of biofilms have complementary metabolic strategies for obtaining nutrients and degrading host immune molecules [14]. Bacteria that reside within mature biofilms are highly resistant to many traditional therapies.

Presently, maggot debridement therapy (MDT) has attracted much attention due to its successful application and efficacy in the elimination of multidrug resistant wound pathogens. In the last few years, several comparative clinical trials investigating the efficacy of MDT have been performed [15, 16]. In terms of debridement, MDT is more effective than conventional therapies [15].

Although, the beneficial debridement effect of larval therapy is well documented, the underlying mechanisms of action, particularly antibacterial and antibiofilm effects, have not been fully elucidated. Nevertheless, maggot* Lucilia sericata* excretions/secretions (ES) are effective for the disruption of* P. aeruginosa* and* S. aureus* biofilms [17, 18]; little is known about antibiofilm activity against another predominant wound pathogen such as* Enterobacter cloacae* (*E. cloacae*) and* Proteus mirabilis* (*P. mirabilis*).

Therefore, the aim of the study was to investigate the antibiofilm activity of sterile maggot ES against* E. cloacae, P. mirabilis,* and* S. aureus* biofilms.

## 2. Materials and Methods

### 2.1. Bacterial Strains

Bacterial isolates* S. aureus* 1141,* E. cloacae* 2383/10, and* P. mirabilis* 719/10 from nonhealing wounds were collected from the Department of Clinical Microbiology in Liptovsky Mikulas Hospital (Liptovsky Mikulas, Slovakia) and Prievidza Hospital (Bojnice, Slovakia). The isolates were transported to the Department of Microbiology, Faculty of Medicine, Slovak Medical University (Bratislava, Slovakia).

### 2.2. Preparation of Sterile and Nonsterile* L. sericata* Larvae

*L. sericata* flies were maintained at the Institute of Zoology SAS under the constant conditions according to [19]. The oviposited eggs were divided into two groups. The first group consisted of sterilized eggs in order to obtain sterile larvae (producing sterile ES products) while the second group consisted of nonsterile eggs to hatch nonsterile larvae producing nonsterile ES products. In the sterile group, the eggs were incubated on the blood agar plate until the larvae reached the third instar stage, where sterile ES products were collected. In the second group, the eggs were incubated on a beef liver; nonsterile larvae were also incubated until they reached the third instar, where nonsterile ES products were collected.

### 2.3. Maggot ES Collection and Preparation

Maggot ES products were collected according to van der Plas et al. [20] with minor modifications. Briefly, the third instar nonsterile and sterile larvae were washed and incubated in Milli-Q ultrapure water for 60 min (50 larvae/200 *μ*L water) at 4°C in a dark place. The incubation was performed at 4°C due to prevention of proteolysis. After incubation, the generated ES products were transferred into a new tube, centrifuged (20 000 g, 4°C, 30 min) to remove solid parts, and finally freeze-dried. Lyophilized ES products were dissolved in Tryptic Soy Broth (TSB) medium (Oxoid, UK) to a final concentration of 100 mg/mL, filtered, and stored at −20°C. The subsequent concentrations of ES products were used in the study: 5 mg/mL, 7.5 mg/mL, 10 mg/mL, 20 mg/mL, 50 mg/mL, and 100 mg/mL. Heat-treated ES (HES) was prepared by incubating native ES products at 100°C for 10 min, centrifuged, and transferred into a new tube.

### 2.4. Determination of ES Influence on Bacterial Growth

An overnight culture of wound isolates was suspended in phosphate buffered saline (PBS) buffer (pH 7.2); the suspension turbidity was adjusted to a 10^8^ colony forming unit (CFU)/mL and was diluted with a medium to the final concentration of 10^6^ CFU/mL. Ten *μ*L aliquots of suspension were inoculated in each well of sterile 96-well polystyrene plates and supplemented with 90 *μ*L of sterile medium; the medium was diluted with ES and HES products. Plates were incubated at 37°C for 22 h; during this period, the bacterial growth was determined by monitoring the absorbance at 570 nm. Adhesive foils (Sigma Aldrich, Germany) were used to avoid plate contamination during the examination of absorbance in a plate reader (Bio-Rad Laboratories, Hercules, CA, USA).

### 2.5. Microtiter Plate Biofilm Formation Assay

Biofilm formation assays were performed according to Stepanovi$\overset{´}{\text{c}}$ et al. [21] using plastic 96-well tissue culture microtiter plates (Sarstedt, Germany). Briefly, a loop full of cells from a blood agar plate was transferred to a sterile 15 mL polystyrene tube containing 4 mL of PBS. The cells were dispersed with a vortex for 1 min and suspension was passed through a 5 *μ*m pore-size syringe filter to remove large clumps of cells. The suspension turbidity was adjusted to 10^8^ CFU/mL and diluted with TSB broth to a final concentration of 10^5^ CFU/mL. Aliquots of bacterial suspension (10 *μ*L) were transferred to each well of the microtiter plate. After incubating bacterial cultures for 24 h at 37°C, the content of each well was aspirated and wells were washed three times with sterile PBS. The remaining attached bacteria were fixed with 100 *μ*L methanol for 15 min. The plates were then stained with 100 *μ*L of 2% (w/v) crystal violet. Afterwards, the dye bound to the adherent cells was resolubilized with 100 *μ*L of 33% (v/v) glacial acetic acid. The optical density (OD) of each well was measured at 570 nm using an automated microtiter plate reader.

### 2.6. Biofilm Inhibition Assay

Biofilms were cultured for 24 h as described above, but 10 *μ*L aliquots of bacterial suspension were inoculated in each well of sterile 96-well polystyrene plates and were supplemented with 90 *μ*L of ES and HES diluted in TSB broth in a range of 5–100 mg/mL. After the incubation time, the remaining attached bacteria were quantified as described in the previous section.

### 2.7. Biofilm Disruption Assay

Biofilms were cultured in a 96-well microtiter plate as described above, with modification according to Stepanovi$\overset{´}{\text{c}}$ et al. [21]. Briefly, after incubating bacterial biofilms for 7 h at 37°C, the content of each well was aspirated; the wells were washed three times with sterile PBS, treated with 100 *μ*L ES and HES products diluted in TSB broth (concentration range of 10–100 mg/mL), and cultivated for an additional 16 h at 37°C. Control biofilms were cultured in TSB broth alone. After the incubation period, biofilms were quantified by CFU enumeration and crystal violet staining as described above. In the case of CFU enumeration, biofilms were rinsed three times; adherent bacteria were detached using a swabbing technique. A cotton-tipped swab was then transferred to 5 mL of PBS buffer and mixed by vortex agitating for 60 s. The bacterial suspensions of* E. cloacae *and* S. aureus* were serially diluted and spread on blood agar plates. In the case of* P. mirabilis* MacConkey agar plates (Oxoid, UK) were used. The plates were incubated at 37°C for 24 h.

### 2.8. Biofilm and Proteinase K Treatment

We examined the effect of proteinase K on biofilm development, on the disruption of established biofilm, and on the cell viability within the biofilm as a positive control and compared potential maggot ES proteolytic activity.

### 2.9. Determination of Protease Activity

Proteolytic activity assay was performed according to [22], using skim milk agar plates. Briefly, 1 g of bacto-agar (Oxoid, UK) and 10 g nonfat dried milk (Sigma Aldrich) were dissolved in 100 mL distilled water and sterilized. Five *μ*L of ES and HES products in range of 5–100 mg/mL was added in a hole in skim milk agar plates and incubated at 37°C for 24 h. Proteinase K at concentration range from 0.1 to 10 *μ*g/mL was used as positive control.

### 2.10. Purification of Antibiofilm Compounds from Maggot ES Products

Potential maggot antibiofilm compounds were purified using a solid phase extraction (SPE) methodology according to [23] with minor modifications. SPE was performed using the column Maxi-Clean SPE C18 600 mg (Grace, USA). One hundred mg of ES products was diluted in 50 mL Milli-Q ultrapure water and centrifuged (20 000 g, 4°C, 15 min) to remove solid parts. The SPE column was conditioned with 10 mL acetonitrile (Sigma Aldrich, Germany) and washed with 10 mL distilled water before loading the sample. It was then washed with 10 mL distilled water and eluted with 5 mL acetonitrile. Final eluate was lyophilized and dissolved in TSB medium to a concentration of 35 mg/mL and tested for protease and antibiofilm activity against* S. aureus* and* E. cloacae* as described above.

### 2.11. Chromatography Fractionation

In order to identify the antibiofilm components of ES products, SPE eluate was fractionated using a reverse phase (RP)-HPLC chromatography (Beckman System Gold, USA) coupled with a C18 column (250 mm × 4.6 mm; 5 *μ*m) (Grace, USA) at a flow rate 0.3 mL/min, by using a gradient from 0 to 90% (v/v) acetonitrile (containing 0.1% (v/v) trifluoroacetic acid), during 70 min. The fractions were lyophilized and dissolved in 100 *μ*L of TSB and tested for antibiofilm activity against* S. aureus.* The active fraction was then fractionated using a C4 column (250 mm × 4.6 mm; 5 *μ*m) (Grace, USA) and tested under the same conditions. The purity of active fractions was checked by electrophoresis on 16.5% Tricine-SDS-PAGE gel using a Mini-Protean II electrophoresis cell (Bio-Rad, CA, USA). Protein bands were detected after staining with Coomassie Brilliant Blue R-250.

### 2.12. Statistical Analysis

Results are presented as the mean with standard error (SEM). All data were statistically analysed from three independent experiments using a Student's *t*-test to determine whether there were significant differences between each ES/HES treatment and untreated control. *P* ≤ 0.05 was considered to be significant. Analyses were performed using GraphPad Prism (GraphPad Software Inc., USA).

## 3. Results

### 3.1. The Effect of Maggot ES on Biofilm Formation of Wound Isolates

**Figure 1:**
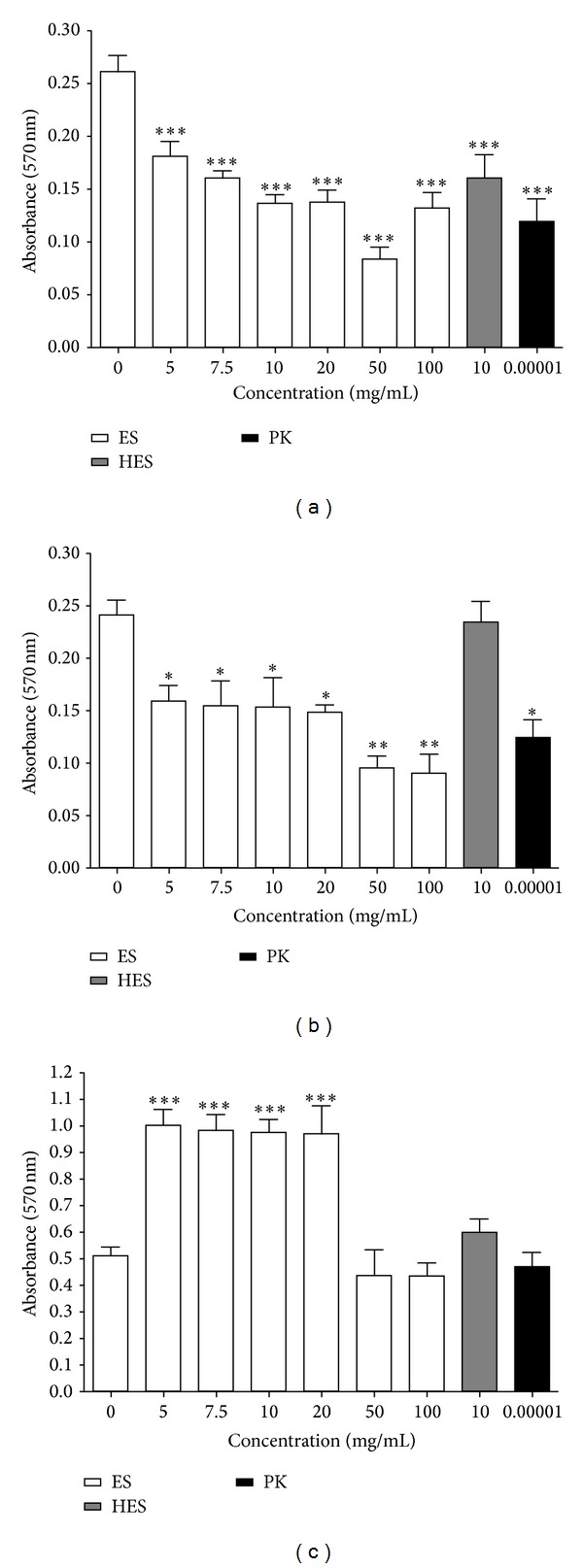
Effects of maggot ES, HES at concentration of 10 mg/mL, and proteinase K (PK) at concentration of 0.01 *μ*g/mL on (a)* Staphylococcus aureus*, (b)* Enterobacter cloacae,* and (c)* Proteus mirabilis* biofilm formation in Tryptone Soya Broth medium after 24 h in 96-well plate. The values of absorbance are mean SEM of three independent assays. The data were statistically analyzed by the Student's *t*-test (*P < 0.05, **P < 0.01, ***P < 0.001).

The ability of maggot ES products to inhibit biofilm formation of* E. cloacae*,* S. aureus,* and* P. mirabilis* was assessed in a 96-well microtiter plate. Maggot ES products with concentration gradient 5–100 mg/mL and HES at concentration of 100 mg/mL did not significantly affect the growth of planktonic bacterial cells of tested isolates.  Figure 1 shows significant inhibition of biofilm formation of* E. cloacae* and of* S. aureus* in the presence of maggot ES compared to control. Biofilm formation was averagely inhibited by 47% in* S. aureus* and by approximately 45% in* E. cloacae* isolate. The most effective maggot ES concentration which inhibited biofilm formation in* S. aureus* was 50 mg/mL. The maggot ES concentration of 100 mg/mL was observed as the most effective in* E. cloacae* where 63% decrease in biofilm formation was observed.

In case of* P. mirabilis,* the side effect of maggot ES was observed. A statistically significant stimulation of* P. mirabilis* biofilm formation was documented in the presence of maggot ES at concentration range 5–20 mg/mL. In order to partially identify the components responsible for the antibiofilm effect of maggot ES, HES was used. HES did not affect biofilm formation of* E. cloacae* and* P. mirabilis,* while in the case of* S. aureus*, HES caused a 51% reduction of biofilm formation. To determine the role of heat unstable ES compounds such as larval protease(s) in inhibition process of biofilm formation, we compared the effect of proteinase K with maggot ES treatment.* E. cloacae* and* S. aureus* exhibited reduction of biofilm formation comparable with ES treatment when treated with proteinase K. The biofilm formation of* P. mirabilis* was unaffected by proteinase K.

### 3.2. The Disruption of Established Biofilm of Wound Isolates by Maggot ES

**Figure 2:**
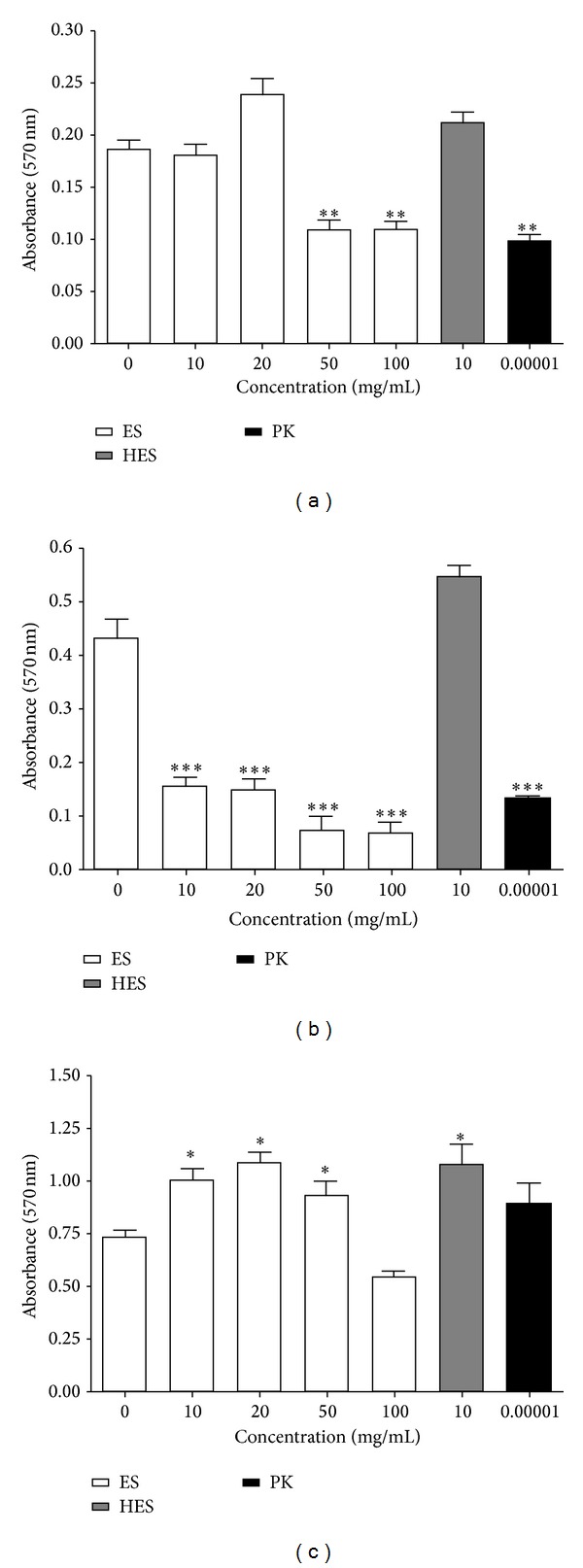
Effects of maggot ES, HES at concentration of 10 mg/mL, and proteinase K (PK) at concentration of 10 mg/mL on established biofilms of (a)* Staphylococcus aureus*, (b)* Enterobacter cloacae,* and (c)* Proteus mirabilis.* Biofilms of wound pathogens were grown in Tryptone Soya Broth medium for 7 h in 96-well plate and treated with maggot ES, HES, and proteinase K for additional 24 h. The values of absorbance are mean SEM of three independent assays. The data were statistically analyzed by the Student's* t*-test (*P < 0.05).

The positive clinical outcomes of MDT suggest that maggot ES products are able to disperse the matured wound biofilm. We determined the ability of maggot ES and HES to detach already established* S. aureus, E. cloacae,* and* P. mirabilis* biofilms. We found that established biofilm of* E. cloacae* was nearly fully disrupted (95% reduction) in the presence of maggot ES at concentration 100 mg/mL and concentration 50 mg/mL partially disrupted (56% reduction) in the case of* S. aureus* compared to the control biofilm (Figure 2). On the other hand, no decrease in* P. mirabilis* biofilm mass in the presence of maggot ES was observed. Furthermore, maggot ES at lower concentrations (10 and 20 mg/mL) significantly increased biofilm mass of* P. mirabilis*. HES had no antibiofilm activity against matured biofilm of* S. aureus* and* E. cloacae*, while it increased the biofilm mass of* P. mirabilis*. The potential biofilm-disrupting trend of termolabile ES products was confirmed by proteinase K. Proteinase K significantly decreased* S. aureus* and* E. cloacae* biofilm mass comparable to observed maggot ES effects.

### 3.3. Maggot ES Products Kill the Cells within the Bacterial Biofilm

**Figure 3:**
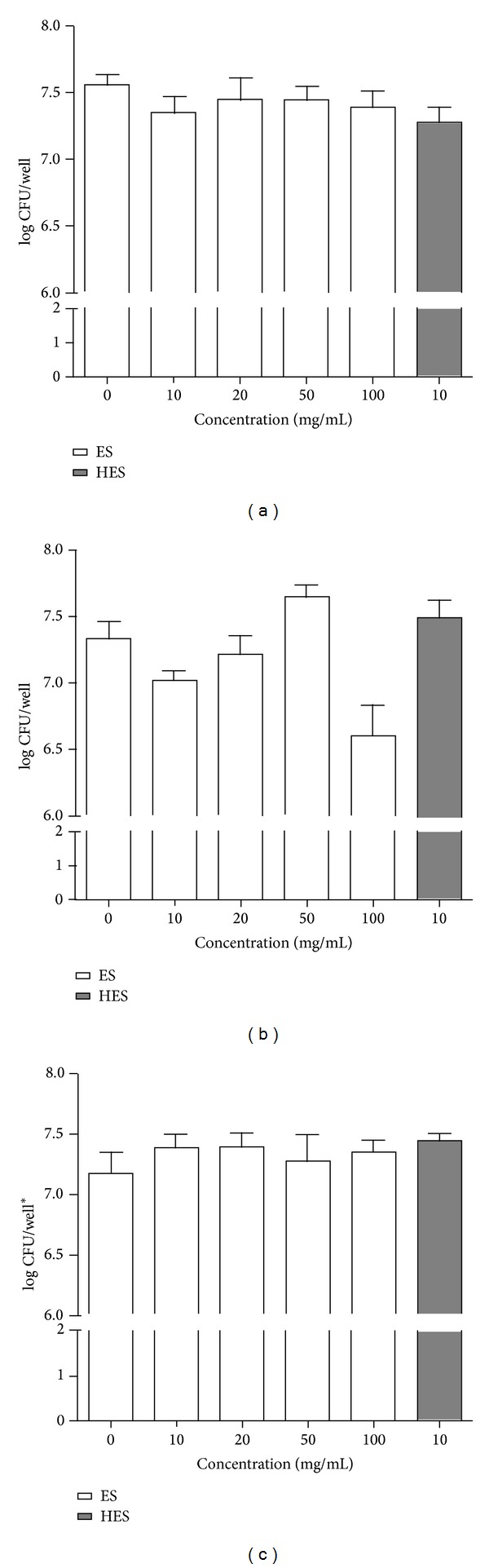
The viable bacteria number in maggot ES and HES treated (a)* Staphylococcus aureus*, (b)* Enterobacter cloacae,* and (c)* Proteus mirabilis* biofilms by log of colony forming unit (CFU) enumeration. The values are number of log CFU per well SEM of three independent assays.

We investigated the maggot ES ability to kill bacteria within the established biofilm of* E. cloacae*,* S. aureus,* and* P. mirabilis*. Already established biofilm was treated with either maggot ES at concentrations of 10, 20, 50, and 100 mg/mL or HES for an additional 17 h. Afterwards we determined the number of viable bacteria (CFU) within the biofilm (Figure 3). Quantification of biofilm formation by CFU enumeration revealed that* S. aureus*,* E. cloacae,* and* P. mirabilis* isolates produced about 10^7^-10^8^ biofilm cells per well. Treatment of* S. aureus* and* P. mirabilis* biofilms with maggot ES did not result in a significant decrease in CFU/well values in log units compared with nontreated biofilms. The killing activity of maggot ES was significant only in the case of* E. cloacae *biofilm; maggot ES decreased the number of viable biofilm cells within a concentration of 100 mg/mL by 10.1% in CFU/well values in log units. Samples treated with heated ES did not show a significant difference in CFU/well values in log units compared with control.

### 3.4. Determination of Protease Activity in Maggot ES

**Figure 4:**
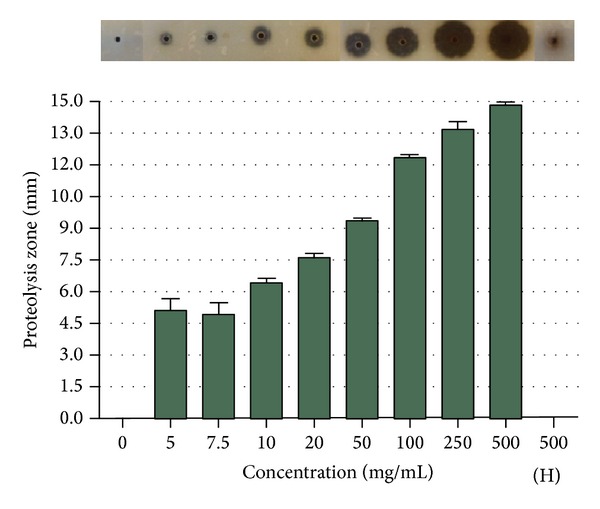
The determination of proteolytic activity in maggot ES products. Maggot ES at concentration range 5–500 mg/mL showed high proteolytic activity which could be observed as a circle zone in the milk agar plate. Heat-treated maggot ES at concentration of 500 mg/mL (500(H)) showed no proteolytic zone.

We investigated the maggot ES proteolytic activity using protease activity assay on milk agar plates. As expected, maggot ES exhibited high proteolytic activity, which could be observed as clear zone by degrading milk proteins. The zone dimension increased with an increasing concentration of maggot ES (Figure 4). There was no visible zone when HES was used. As a positive control we used proteinase K.

### 3.5. Partial Purification of Antibiofilm Compound(s) from Maggot ES Products

**Figure 5:**
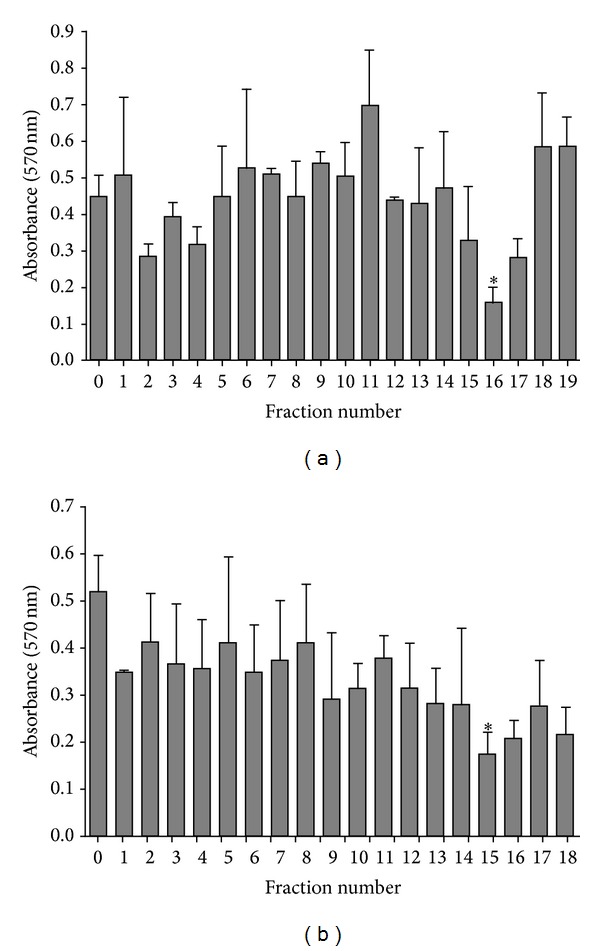
Chromatography fractionation of maggot ES SPE eluate. Maggot ES SPE eluate was fractionated by HPLC system using C18 column (250 mm × 4.6 mm; 5 *μ*m) at a flow rate 0.3 mL/min, by using a gradient from 0 to 90% (v/v) acetonitrile (containing 0.1% (v/v) trifluoroacetic acid), during 70 min (a). Fractions with antibiofilm activity against* S. aureus* established biofilm were then fractionated by C4 column with the same conditions (b) and checked for its activity against* Staphylococcus aureus* established biofilm. PC is positive control, established biofilm without ES treatment.

**Figure 6:**
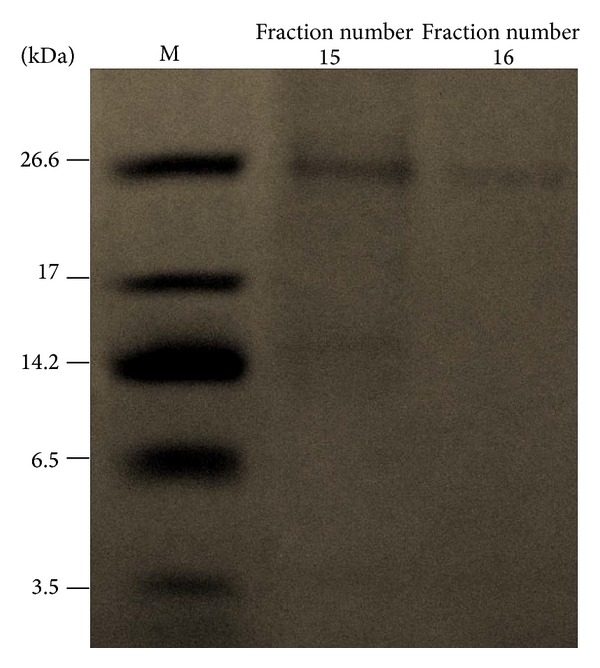
The size (kDa) of partially purified antibiofilm fraction against* Staphylococcus aureus* isolate. Fraction numbers 15 and 16 were done on Tricine SDS-PAGE. The gels were stained by Coomassie Blue-R. The active band was about 25 kDa in size, present in fraction number 15 and fraction number 16, but in the case of fraction number 16, the band is clearer.

The first purification step of maggot antibiofilm compounds was based on the use of SPE-C18 cartridge. The obtained eluate was tested against established biofilm of* S. aureus* and* E. cloacae*. The results showed strong antibiofilm activity against matured biofilm. It decreased the* S. aureus* and* E. cloacae* biofilm by 74% and 83% in eluate fraction. Afterwards, SPE-C18 eluate was fractionated in HPLC system using C18 column (Figure 5(a)). The obtained fractions were tested for activity against* S. aureus* established biofilm. The fraction with retention time between 56.6 and 60 min (fraction nos. 15 and 16) degraded matured biofilm by 73%. This active fraction was then fractionated on C4 column employing the same conditions (Figure 5(b)). The fractions with retention time 54–61 min (fractions nos. 15 and 16) significantly degraded* S. aureus* established biofilm by 76 to 71%. The antibiofilm fractions contained a protein with MW of around 25 kDa (Figure 6).

## 4. Discussion

Bacterial biofilms formed by pathogenic bacteria cause serious troubles in the human health and delay wound healing process [23]. We investigated the antibiofilm activity of maggot ES against wound isolates* S. aureus*,* E. cloacae,* and* P. mirabilis*, some of the most clinically relevant species. Our results suggested that maggot ES prevented biofilm formation of* S. aureus* and* E. cloacae* but stimulated biofilm formation of* P. mirabilis*. Maggot ES treatment of preformed bacterial biofilms revealed differences in the ability to disrupt biofilm and kill the bacteria within* S. aureus* and* E. cloacae* biofilms. In case of* P. mirabilis*, we observed that maggot ES increased biofilm mass and exhibited no effect on cell viability in biofilm. There are a limited number of studies that attempted to elucidate the effect of maggot ES on the ability of bacteria to form biofilm communities and the potential use of ES as an agent to disrupt existing bacterial biofilms [17, 24–27].

Maggot ES are continuously secreted/excreted to the wounds by* L. sericata* larvae during the MDT. Antibiofilm action of maggot ES depends on the wound characteristics and the wound bacterial environment [28]. Based on our results, maggot ES may either reduce or stimulate biofilm formation in wound depending on the presented bacterial strain. Furthermore, maggot ES-induced reduction and disruption of bacterial biofilm depend on the concentration of active substances.

According to the previous studies of maggot ES antibiofilm activity [17, 26] we confirmed that maggot ES contain substances able to prevent biofilm formation and disrupt established biofilm of* S. aureus.* In our study, we report strong antibiofilm activity of maggot ES against wound pathogen* E. cloacae*. Interestingly, maggot ES were more effective in preventing the biofilm formation and dispersing the matured biofilm of* E. cloacae* than the biofilm of* S. aureus*. Also, we observed that maggot ES were able to significantly affect the cell viability within the biofilm of* E. cloacae* while it failed in the case of* S. aureus*. The present data confirm the fact that maggot ES are differentially effective against different bacterial species. Our results indicate that maggot ES eradicate the bacterial biofilm of different bacterial strains through different mechanisms.

In the present study, maggot ES induced the elimination of* S. aureus* biofilm mass but the number of viable cells was not significantly decreased. In case of* E. cloacae* we observed complete disruption of the preformed biofilm and significant decrease of viable cells within the biofilm. It is observed that the antibacterial maggot ES compounds were able to diffuse and kill the bacterial cells through the established biofilm matrix. Heat-treated maggot ES lost antibiofilm activity against* E. cloacae* but remained in the inhibition of forming* S. aureus* biofilm. This shifted the attention to heat-labile compounds of maggot ES such as larval proteases.

We found that maggot ES exhibited high proteolytic activity. Proteases play an important role in biofilm regulation of gram-positive and gram-negative bacteria [29]. Maggot ES contain a mix of different extracellular proteases. These proteases play a crucial role in cleaning the wound bed of chronic wounds during MDT [30, 31]. Three groups of proteolytic enzymes (metalloproteinases, serine and aspartyl proteases) were identified in the maggot ES products [30]. In addition, we have very recently identified five novel putative proteases of* L. sericata* maggots and demonstrated that they could be secreted into the wound during the MDT [32].

The importance of antibiofilm properties of heat sensitive compounds within maggot ES which may be responsible for breaking down* S. aureus* biofilm was discussed in previous studies [20]. Brown and coworkers [18] found that nucleases present in maggot ES can digest DNA associated with* P. aeruginosa* biofilm formation. In a very recent study it has been presented that maggots chymotrypsin disrupts protein-adhesin mediated biofilm formation of* S. aureus* [33]. Van der Plas and coworkers [27] indicated that potential molecules in maggot ES responsible for breaking down* S. aureus* biofilm are proteases belonging to the group of serine proteases. Harris and coworkers identified a molecule with antibiofilm activity against* S. epidermidis* biofilm; the particular responsible molecule(s) was >10 kDa in size and appeared to have protease or glucosaminidase activity [25]. In the present paper we also partially purified a compound of maggot ES with antibiofilm properties against* S. aureus* isolate with a size of around 25 kDa. However, the exact structure of the molecules responsible for the antibiofilm properties of maggot ES is yet unknown.

Interestingly, maggot ES in particular concentrations stimulated biofilm formation as well as significantly increased the biomass of* P. mirabilis* preformed biofilm. The number of viable cells within* P. mirabilis* biofilm was not affected after maggot ES treatment.* P. mirabilis* is a commensal organism living in digestive system of dipteran larval stage [34]. This symbiotic bacterium adheres to peritrophic membrane with the help of lectins and it is important for maggots surviving in their natural pathogenic environment [35]. The contaminated food is easily ingested and microorganisms are rapidly eliminated due to interaction with substances produced by* P. mirabilis* [36]. One of the beneficial substances produced by* P. mirabilis* is mirabilicide [37] and other diverse proteolytic enzymes [25, 38, 39]. These compounds contribute to protect the larvae from other harmful bacteria in food intake [36]. This relationship may explain why maggot ES did not show any antibiofilm activity against* P. mirabilis*.

In conclusion, maggot ES products were shown to be effective in the reduction of biofilm formation and the eradication of established biofilms of* E. cloacae* as well as* S. aureus* wound pathogens. The antibiofilm effects of maggot ES are mainly mediated by proteases. On the other hand, maggot ES protected or stimulated* P. mirabilis* biofilm formation. Therefore, MDT could be used as a potential therapy for the treatment of wounds containing* E. cloacae* and* S. aureus* but not for* P. mirabilis*.
